# Synopsis of the Mütter Lectures

**Published:** 1891-02

**Authors:** Herbert Upham Williams


### Correspondence.

---

*Dr. H. U. Williams gives a Synopsis of the Mütter Lectures Delivered in Philadelphia by Dr. Roswell Park.*

*Editors Buffalo Medical and Surgical Journal :*

It has occurred to me that the profession of Buffalo and vicinity will be interested in an account of the Mütter lectures, which are being delivered this Winter by Dr. Roswell Park.

Dr. Mütter, of Philadelphia, left, by his will, a sum of money to maintain a course of lectures upon some topic relating to surgical pathology. They are given every third year. The first six of Dr. Park's papers have already been read at the building of the College of Physicians, before a large attendance of the medical men of this city, beginning on the evening of December 4th.

I. The first portion was introductory, sketching the progress of surgical pathology, especially as relating to the study of microörganisms, and eulogistic of the scientists who have achieved the brilliant results of recent times in this direction.

A brief account of inflammation followed. Particular attention was given to the granulomatous form, and much stress was laid upon the influence of constitutional tendency towards it. The phenomena of thrombosis were treated of as being closely connected with inflammation. Ptomaines and leucomaines were shown to have a powerful influence upon the blood, and to be partly responsible for the alterations seen in it after death by the infectious diseases.

Much interest was found in the study of traumatic anemia, especially in the practical bearing of the observations of Mikulicz. A standard of 100 per cent. of hemoglobin was established by examination of blood from healthy young men. This standard was discovered not to be attained in old age, in childhood, nor in females. It was also determined that the greatest reduction consistent with life was to about twenty per cent. It was the opinion of Mikulicz that many deaths after operation are from oligochromemia. It was considered possible that estimation of the amount of hemoglobin in a patient might furnish a pretty accurate indication as to whether or not an operation could be borne.

At the same time, authority was given, showing one-fifth of the body to be anaërobic. Metabolism was none the less regarded as taking place in all parts. This led to a discussion of the products of cell activity in the body, and their resemblance to those of microörganisms. A ptomaine was defined as the product of cells introduced from without, and as especially connected with putrefactive change; a leucomaine, as the result of the destruction of albuminoids by the cells of the living organism. Cultures were exhibited showing the formation of ptomaines by Poehl's test, (.05 per cent. perchloride of iron, mixed with gelatine, staining occurring at line of contact). A classification and list of ptomaines, with the origin of each, were given.

All this was preparatory to the subject proper of the course,—the surgical infectious diseases. Predisposition to infection was discussed, and illustrated by the immunity of certain animals to particular bacteria, and, in the same connection, the strongly resistant properties of some tissues and organs, and comparatively feeble of others.

The lecture closed with a consideration of embolism, general or local depression of vitality, inflammation, cold, and injury as predisposing to infection.

II. The second lecture continued this subject. The point of inoculation was shown to be of much importance. Organisms that might thrive if introduced into certain tissues, would die in some others. The pyogenic cocci were said to grow best in cellular connective tissue. Diabetes has long been known to dispose to suppurative inflammations. Explanation was offered in the fact that the Staphylococci are favorably influenced by grape sugar.

In relation to susceptibility it was further stated that a sufficiently large amount of virus might affect a comparatively insusceptible animal.

The effect of one bacterium on the growth of another was declared to depend on what the two forms were. Obviously an aërobic colony might foster an anaërobic. But some species were inimical to others, as the micrococcus of erysipelas to the bacillus of anthrax.

The question of an exact definition for the term pus was elaborately discussed. Virchow's and Cohnheim's views in regard to inflammation were expounded, and a middle ground between them advised. Metschnikoff's doctrine of phagocytosis was reviewed. It was admitted that simple irritation might result in an exudate closely resembling pus, and that similar discharges have daily been observed from aseptic wounds; furthermore, that the pus of cold abscesses was often destitute of microörganisms, though they had probably been present at an earlier period. Experiments were cited in which cadaverin was injected into the tissues of lower animals. A high degree of inflammation followed the irritation, and an exudate, similar to pus, but devoid of bacteria. Nevertheless, it was decided that the microörganism should be regarded as an essential feature of true pus. For pus-like exudates, not due to bacteria, the term *puruloid* was suggested; and *archepyon*, for pus such as that from cold abscesses, whose microbes had been destroyed in their own product. *Pyophylactic* was offered as a substitute for *pyogenic*, referring to the membrane lining abscess cavities, as being more expressive of its true function.

III. The third lecture was given up to a description of the pyogenic organisms. The terms, obligate and facultative, were defined as referring to the invariable or occasional production of pus respectively. The following list of pyogenic microörganisms was given: Obligate.—(1). Staphylococcus pyogenes aureus, (2). S. pyogenes citreus, (3). S. pyogenes albus, (4). S. cereus albus, (5). S. cereus flavus, (6). S. flavescens, (7). S. pyosepticus, (8). Streptococcus pyogenes, (9). Bacillus pyocyaneus, (10). B. pyogenes fœtidus. Facultative.—(1). Gonococcus, (2). Pneumonococcus (of Fränkel), (3). Pseudopneumonococcus, (of Passet), (4). Micrococcus pyogenes tenuis, (5). Rosenbach's oval coccus, (6). Bacillus malleí (of glanders), (7). Vibrion septique (of malignant edema), (8). Bacillus tuberculosis, (9). B. tetani, (10). B. of typhoid fever, (11). B. anthracis, (12). Micrococcus tetragonus, (13). Actinomyces, (14). Aspergilli (of several species). As to the following there seemed to be some question: Bacillus gangrenæ, Micrococcus fetidus, M. of Delhi boil, Amoeba of dysentery.

The properties of these bacteria were treated of in too much detail to admit of condensation. The status of the coccus of erysipelas was regarded as somewhat doubtful. Cerebro-spinal meningitis was stated to be caused by a number of different forms.

IV. The fourth evening was devoted to the study of sepsis. In connection with it the following conditions were considered:

(1). Surgical fever,—the direct consequence of the injury, and not due to infection. (2). Intestinal toxemia,—poisoning by ptomaines or leucomaines, developed by bacteria in the alimentary canal, or as a result of derangement of the digestive processes. (3). Sapremia,—ptomaines absorbed from some cavity, bacterial growth not extending into the tissues, as, decomposition of clots in the puerperal uterus, or between the flaps of an amputation stump. (4). Septicemia,—an extension of the last form, the microörganisms having invaded the contiguous structures. (5). Pyemia,—further progression by an infective embolic process, with the formation of multiple abscesses.

The means of protection possessed by the body were found to lie in the fact that highly albuminous liquids retard the growth of microbes, in the property of phagocytosis, and in the peculiar preferences of some bacteria for certain tissues, while not thriving in others.

Ptomaine poisoning, such as intestinal toxemia, already existing, favored the development of microörganisms. Laboratory experiments were described, showing remarkable variations from clinical results. Cocci, injected into the circulation in healthy animals, usually failed to develop, even if some wound were made, and often when directly applied to it. Recovery from the wound took place in some cases before there was time for their growth. The query was made: "What kind of a local injury, then, is needed?" It was suggested that perhaps too much importance had been attached to the bacteria alone.

V. Tetanus was the topic of the fifth lecture, being preceded by a consideration of tetany as preparatory to it. Tetany was described as a spasmodic condition of nervous origin, usually manifested in the muscles of the face and arms, less commonly in the legs, consciousness seldom being lost, not being connected with infection or suppuration, and sometimes fatal in its results. Exposure, intestinal irritation, and other causes were mentioned, but its chief interest was found in relation to thyroidectomy. Of late years many cases of tetany have been reported as following this operation for the relief of goitre. Apparently, in such instances, tetany took the place of myxedema. Curious discrepancies were discovered in the proportion of cases of tetany and myxedema occurring in the hands of different surgeons.

Experiments on lower animals showed that loss of the thyroid gland usually caused tetany in carnivora, but not in herbivora.

Transplantation of the thyroid to the peritoneum was successful a few times in averting tetany.

Tetanus was then taken up, its causes first being considered. While it usually followed a wound, preferably lacerated, yet solution of continuity on the surface was not absolutely necessary, instances having been known where it ensued upon dislocation and contusion. Circumstances were cited that sometimes operate as causes,—for instance, a hot climate. A large proportion of cases occurred among those working about horses. Tetanus hydrophobicus and neonatorum were regarded as true tetanus.

It has been within the last ten years that the microbe of tetanus has been isolated by Nicolaier, and carefully studied by Kitasato. It was described as an anaërobic bacillus, having spores that are extremely resistant, withstanding five per cent. carbolic for ten hours. Similar organisms were discovered in the soil, explaining the so-called "telluric" origin of tetanus.

Dr. Park believed them to flourish in the vicinity of horses. Remarkable difficulty was encountered in finding the bacillus after it had been introduced experimentally into lower animals, even in the neighborhood of the wound. None the less, tetanus developed in spite of cauterization or excision of the structures about the point of inoculation. A tube was exhibited, showing a colony of the bacilli flourishing beneath the surface of the culture medium.

Tetanine and tetanotoxine were mentioned as alkaloidal substances, which have been isolated from cultures of the fungi. The symptoms produced by them were like those of tetanus, but were only manifested after doses so large that some more virulent poison must also be supposed to exist.

VI. The study of peritonitis was prefaced with reports of experiments showing the healthy peritoneum capable of absorbing considerable amounts of fluid. Furthermore, comparatively large numbers, even of the pyogenic cocci could be disposed of. This would not obtain, however, if a certain limit were passed. Departures from the normal state might overcome the resisting power; as, for instance, presence of ascitic fluid, which furnished a good culture medium. Obstructions to absorption, presence of blood clot, the action of irritants, and local injury were mentioned as among the conditions permitting the growth of bacteria. In laparatomies, microörganisms were supposed always to enter the peritoneum, but under normal conditions they should be destroyed.

The classification of the different forms of peritonitis accepted was: aseptic, purulent (chiefly from staphylococci or streptococci), putrid (mixed infection), and specific (including tubercular and gonorrheal). It was questioned whether a purulent peritonitis could follow pure gonorrheal infection.

A brief account was then given of the means employed for learning the powers of antiseptics, detailing, in particular, the methods used in Koch's laboratory. A large number of substances have been tested there, determining the strength of solution and time necessary for them to operate, their action on different microbes, and their poisonous effects upon animals. The antiseptic has yet to be found that will not kill the human organism in one-fifth or one-fourth the strength required to destroy bacteria in it.

Dr. Park stated that he had tested the new antiseptic, pyoktanin, after Koch's method, and found neither the yellow nor the blue reliable as antiseptics, although possessing this property in some degree, as all the aniline dyes probably do.

The remaining four of Dr. Park's lectures will not be read for a number of weeks. HERBERT UPHAM WILLIAMS.

